# Sesquiterpenoid Hormones Farnesoic Acid and Methyl Farnesoate Regulate Different Gene Sets in Shrimp *Neocaridina davidi* Hepatopancreas

**DOI:** 10.3390/biom15060815

**Published:** 2025-06-04

**Authors:** Yehui Luan, Wenyan Nong, Wai Lok So, Jerome Ho Lam Hui

**Affiliations:** School of Life Sciences, Simon F.S. Li Marine Science Laboratory, State Key Laboratory of Agrobiotechnology, Institute of Environment, Energy and Sustainability, The Chinese University of Hong Kong, Hong Kong SAR, China; 1155186094@link.cuhk.edu.hk (Y.L.); nongwenyan@cuhk.edu.hk (W.N.); henrysowailok@yahoo.com.hk (W.L.S.)

**Keywords:** crustacean, shrimp, *Neocaridina*, genome, transcriptome, farnesoic acid, methyl farnesoate, sesquiterpenoid hormone

## Abstract

Sesquiterpenoid hormones such as the juvenile hormone and methyl farnesoate (MF) are well known to respectively control the development and reproduction in insects and crustaceans (such as shrimp, crabs, and lobsters). In recent years, the sesquiterpenoid hormone farnesoic acid (FA) has also been identified in other non-insect/crustacean invertebrates; despite this, their regulatory roles remain poorly understood. Here, we carried out the in vitro treatments of MF and FA on the hepatopancreas of female adult shrimps *Neocaridina davidi*. Transcriptomic analyses revealed a total of 65 and 112 differentially expressed genes in the MF- and FA-treated hepatopancreas at 3 h post-treatment, respectively. Gene pathway enrichment analyses further suggested that the two sesquiterpenoid hormones regulate different sets of genes, with the gene pathway involved in pancreatic secretion enriched only in the FA-treated hepatopancreas. This study demonstrates the differential regulatory roles between sesquiterpenoid forms, which warrants further investigation into the functions of FA in crustaceans.

## 1. Introduction

Arthropods, or jointed leg animals, are the most successful group of animals, accounting for more than 80% of described animal species. Decapod crustaceans are a group of arthropods that include crabs, lobsters, crayfish, and shrimps, and they play important roles in the environment as well as for global food security [1,2,3,4]. Nevertheless, our understanding of the development and reproduction of arthropods largely comes from insects [5,6].

In insects, the sesquiterpenoid hormone juvenile hormone (JH) is well known to control development, reproduction, and metamorphosis [5,7]. Despite JH only being produced in the insects, other forms of sesquiterpenoids, including farnesoic acid (FA) and methyl farnesoate (MF), have also been identified in various invertebrate lineages [8,9,10,11,12]. Since the discovery of MF in crab back in 1987 [13], and later also in various other groups of crustaceans [8,14,15], the functions of MF in different crustacean lineages have been found to be similar to JH in insects, including the regulation of development, physiology, metamorphosis, and reproduction [6,16,17]. Nevertheless, the functions of other sesquiterpenoid forms remain largely unexplored, with limited studies demonstrating in vitro treatments of FA in crab *Charybdis feriatus* and lobster *Homarus americanus* showing the upregulation of vitellogenin/apolipoprotein gene expression [18,19]. Thus, which genes are being regulated by FA in crustaceans remain poorly explored.

The freshwater cherry shrimp *Neocaridina davidi* is a common pet found in various aquarium worldwide, and it has previously been suggested to be a potential decapod crustacean model given its availability, easiness to culture, and relatively short growth rate comparing to other decapod crustaceans [20]. To date, it remains the only decapod crustacean that is feasible for CRISPR mutant production for gene function investigation [21]. Among the various commercially available cherry shrimps, the red color morph is popular not only for pet owners but also for researchers because its transparent body allows for the different gonad stages to be easily seen [20]. In this study, we investigated the genome-wide gene expression in the females’ hepatopancreases after treatment of the sesquiterpenoid hormones FA and MF.

## 2. Methodology

### 2.1. Animal Sampling and Culture

Cherry shrimp *Neocaridina davidi* were purchased from a pet store in Hong Kong SAR and were cultured at 18–25 °C, 12 h light/12 h dark cycle in filtered tap water as previously described at the Chinese University of Hong Kong SAR [22]. The shrimps were ~4–5 months old with body lengths of 1.2–1.4 cm. Seven days prior to the experiment, shrimps were kept in sufficient aeration and circulating water and were fed with a commercial diet each day at around 6 pm. At 24 h prior to the experiment, the shrimps were deprived of food for dissection and hormone treatments.

### 2.2. In Vitro Sesquiterpenoid Hormones Treatment

The hepatopancreases from red color females (with ovaries developed) were dissected, rinsed with 1X PBS, and cultured in the culture medium for 3 h and 6 h at 25 °C. For the control group, 1 mL of 1X Schneider’s *Drosophila* medium (Thermo Fisher, Hong Kong SAR, China) was used as the culture medium. For the FA and MF treatment groups, the culture medium was prepared by adding FA or MF solution to 1 mL of 1X Schneider’s *Drosophila* medium for a final concentration of 1 ppm. A GeneJET RNA purification kit (Thermo Fisher, Waltham, MA, USA) was used to extract RNA from the treated tissues. The hepatopancreases of three shrimp individuals were pooled together for each sample and each group had three samples. The purity of RNA was assessed using a Nanodrop2000 spectrophotometer (Thermo Fisher, Waltham, MA, USA).

### 2.3. Transcriptome Sequencing and Analyses

Extracted RNA at 0 h and 3 h were sent to Novogene Corporation, Inc. (Hong Kong SAR, China) for library construction and sequencing. Each sample was sequenced on the Illumina NovaSeq 6000 platform (PE150), generating 6 GB of raw data. De novo assembly was performed by Trinity (v2.9.1) [23] with default parameters using the transcriptome data processed as described above. For gene annotation, BlastX (v2.12.0) was used to match the transcripts with public databases, including NCBI nr, uniprot, and KEGG databases. The top result of each query with e-value < 1 × 10^−5^ was selected, and all results were summarized using Trinotate (v4.0.2) [24]. Trinity script ‘align_and_estimate_abundance.pl’ was then used for transcript quantification. The R package Deseq2 (v1.44.0) was used to filter differentially expressed genes (DEGs). Genes with q ≤ 0.05 and |log2_ratio| ≥ 1 were identified as DEGs if the conditions that 3 replicates must have cpm values > 0 and average cpm > 50 were fulfilled.

### 2.4. KEGG and Gene Ontology (GO) Enrichment Analyses

KEGG (Kyoto Encyclopedia of Genes and Genomes, http://www.kegg.jp/ (accessed on 13 March 2024)) and GO (Gene Ontology, http://geneontology.org/ (accessed on 4 March 2025)) pathway enrichment were carried out on selected DEGs using the R package ClusterProfiler (v4.83) [25] (*p* < 0.05 was considered to be significantly enriched).

### 2.5. Weighted Gene Co-Expression Network Analyses (WGCNA)

The R package WGCNA (v1.72) was used to identify co-expression gene modules in each treatment [26]. The correlation between genes and sample groups is quantified as Gene Significance (GS), while the average GS for all genes within a module is termed as Module Significance (MS). Genes with GS > 0.2 and MS > 0.8 in the selected module were identified as hub genes.

### 2.6. Real-Time PCR Validation

cDNA was generated from 0.8 μg total RNA of each sample using iScript reverse transcription supermix (Bio-Rad, Hercules, CA, USA). PCR was performed using iTaq™ Universal SYBR^®^ Green Supermix (Bio-Rad, Hercules, CA, USA) according to the manufacturer’s instructions, and processed on the CFX96 Touch Real-Time PCR System (Bio-Rad, CA, USA) in a total reaction volume of 15 μL. The thermal cycling conditions were shown as follow: 95 °C for 3 min, 40 cycles at 95 °C for 5 s, and 55 °C for 30 s. The relative expression level of each transcript was calculated using the 2 −ΔΔCt method. The primers used for real-time PCR validation are shown in Table S1.

## 3. Results

### 3.1. Differential Gene Expression in Hepatopancreas upon FA or MF Treatment

**Figure 1:**
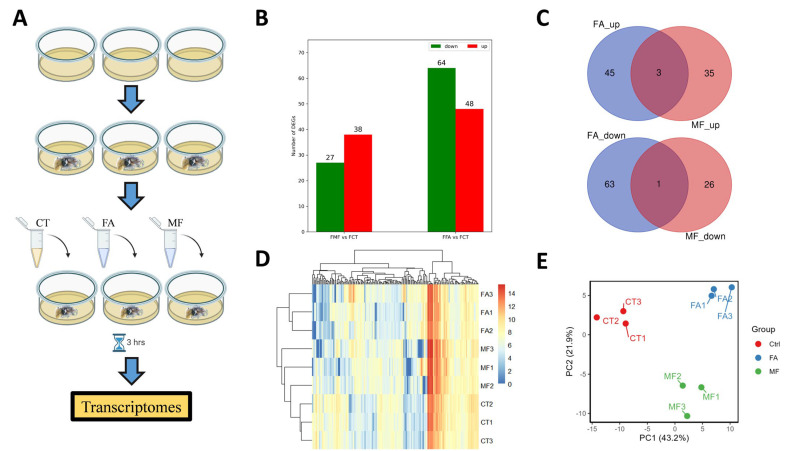
(**A**) Experimental design of this study. (**B**) Number of differentially expressed genes (DEGs) between the controls and sesquiterpenoid hormone FA/MF treatments. (**C**) Venn diagrams of up- and down-regulated DEGs between the controls and FA/MF treatments. (**D**) Heatmap of the DEGs after the sesquiterpenoid hormone FA/MF treatments. (**E**) Principal components analyse (PCA) of the DEGs after the sesquiterpenoid hormone FA/MF treatments.

In vitro sesquiterpenoid hormones FA and MF treatments were performed on the hepatopancreas of mature female *N. davidi*. The experimental design is shown in Figure 1A. cDNA libraries were constructed from nine samples, including the controls, 3 h post-FA treatment, and 3 h post-MF treatment, with each having three biological replicates. The number of clean reads generated in each library is shown in Table 1.

In the de novo transcriptomic analyses, a total of 112 and 65 differentially expressed genes (DEGs) were revealed for FA treatment and MF treatment groups, respectively, when compared to the controls (Figure 1B, Tables S2 and S3). Among these differentially expressed genes, there were only four common DEGs revealed between the FA and MF treatment groups, including filamin-A-like isoform X1, chymotrypsin-1D protein, TRINITY-DN2181-c0-g2-i1, and Carboxypeptidase A1 (Figure 1C). Following treatment, a heatmap of the DEGs was created (Figure 1D) and principal components analyses of the DEGs were performed (Figure 1E).

### 3.2. KEGG Gene Pathway Enrichment Analyses

**Figure 2:**
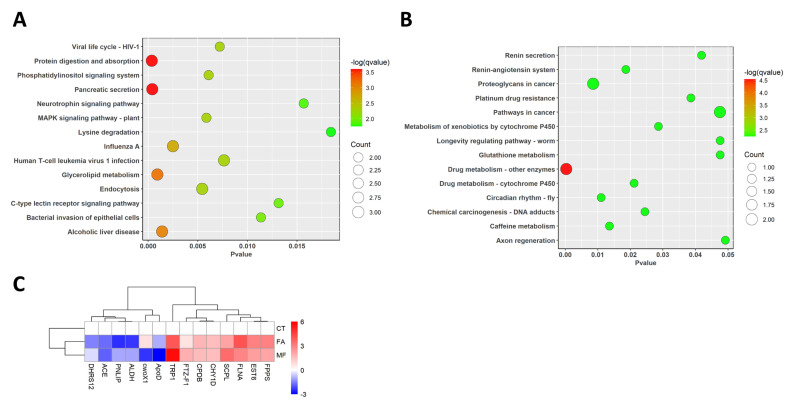
KEGG pathway enrichment analyses of DEGs between (**A**) control and FA treatment groups and between (**B**) control and MF treatment groups. (**C**) Cluster diagram of selected DEGs between controls and each hormone treatment (relative log expression to the control group).

Here, we carried out KEGG gene pathway enrichment analyses in de novo transcriptome analysis. For the DEGs identified in the FA treatment group, they were mainly enriched in 14 gene pathways: protein digestion and absorption, pancreatic secretion, glycerolipid metabolism, alcoholic liver disease, influenza A, endocytosis, the MAPK signaling pathway, the phosphatidylinositol signaling system, the viral life cycle, human T-cell leukemia virus 1 infection, the bacterial invasion of epithelial cells, the c-type lectin receptor signaling pathway, the neurotrophin signaling pathway, and lysine degradation (Figure 2A). Among the above gene pathways, three genes including the *lysosomal Pro-X carboxypeptidase*, *carboxypeptidase B-like*, and *trypsin* genes were differentially regulated in the protein digestion and absorption pathway. For the endocytosis gene pathway, the *WASH complex subunit 5 isoform X1*, *cation-independent mannose-6-phosphate receptor*, and *tumor susceptibility gene 101* proteins were differentially regulated. Furthermore, *pancreatic triacylglycerol lipase*, *carboxypeptidase B-like*, and *trypsin* were differentially regulated in the pancreatic secretion pathway and *calmodulin-like protein 4* and *copper-transporting ATPase 1* were differentially regulated in the MAPK signaling pathway.

Apart from the above gene pathways, two of the genes were revealed to be differentially regulated in the mevalonate (MVA) pathway, i.e., *ALDH* and *FPPS*, with aldehyde dehydrogenases (ALDHs) showing a downregulation trend and farnesyl pyrophosphate (FPPS) showing an upregulation trend in the FA treatment group (shown in Figure 2C).

For the MF treatment group, a total of 14 gene pathways were found to be enriched, including drug metabolism-other enzymes, proteoglycans in cancer, circadian rhythm—fly, caffeine metabolism, the renin–angiotensin system, drug metabolism—cytochrome P450, chemical carcinogenesis—DNA adducts, metabolism of xenobiotics by cytochrome P450, platinum drug resistance, renin secretion, pathways in cancer, glutathione metabolism, longevity regulating pathway—worm, and axon regeneration. Among the above gene pathways, *glutathione S-transferase D7* and *xanthine dehydrogenase* were differentially regulated in the drug metabolism-other enzymes pathway, while *filamin-A-like isoform X1* and *rho guanine nucleotide exchange factor 11 isoform X1* genes were differentially regulated in the proteoglycans in cancer pathway (Figure 2B).

### 3.3. Gene Ontology (GO) Enrichment Analysis

GO enrichment was conducted to identify the biological functions associated with the DEGs. For the FA treatment group, the DEGs were enriched in 64 GO pathways, including 40 pathways in the biological process category, 9 pathways in the cellular process category, and 15 pathways in the molecular function category (Table S4). Among the enriched pathways, 1-phosphatidylinositol 3-phosphate 5-kinase and two isoforms of phosphatidylinositol 3,4,5-trisphosphate 3-phosphatase and phosphatidylinositol 4-kinase type 2-alpha isoform X1 were enriched in the phosphatidylinositol biosynthetic pathway. Two isoforms of the cation-independent mannose-6-phosphate receptor as well as copper-transporting ATPase 1 were also found enriched in the trans-Golgi network transport vesicle pathway. For the MF treatment group, the DEGs were only found to be enriched in three GO pathways: lamellipodium morphogenesis, early endosome to Golgi transport, and the retromer complex (Table S5).

### 3.4. WGCNA for Identification of Hub Genes Responsible for FA and MF Treatment

**Figure 3:**
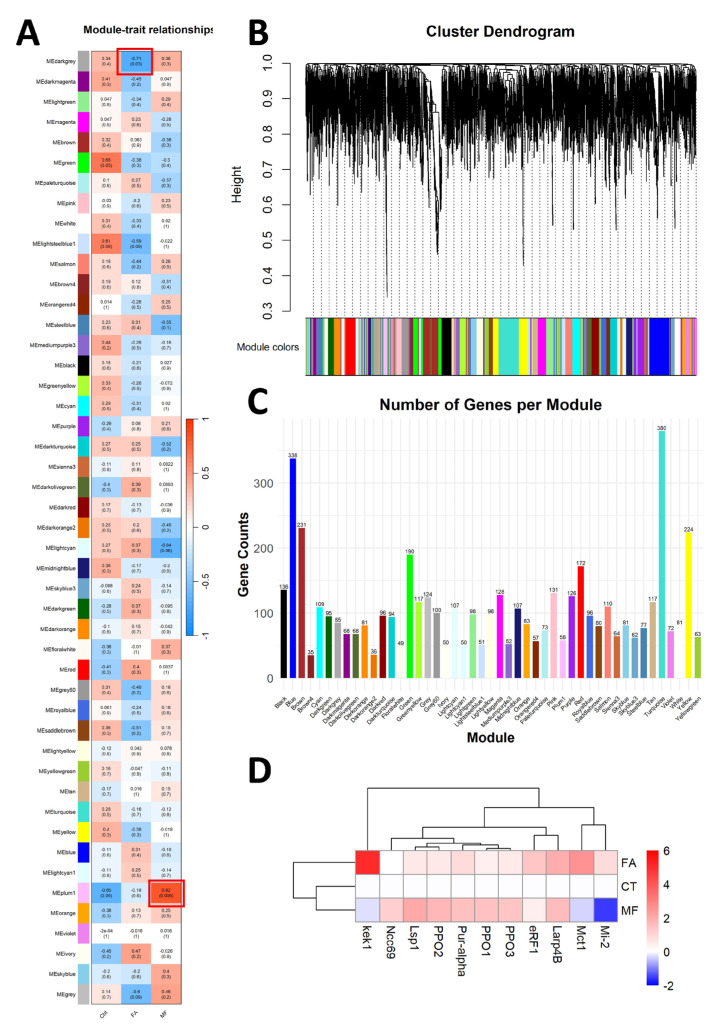
(**A**) Pearson correlation analysis results between each module and treatment. The red box highlights the modules with highest absolute value of Pearson correlations in the FA and MF treatment group. (**B**) Cluster dendrogram of the top 5000 common genes for median absolute deviations in all samples. (**C**) Number of genes in each module. (**D**) Cluster diagram of selected hub genes in module plum1 and module darkgrey (relative log expression to the control group).

The genes of the top 5000 median absolute deviations were selected as input for WGCNA to construct co-expression modules (Figure 3B). As shown in Figure 3A, the genes were clustered into 37 modules, and the number of genes in each module is shown in Figure 3C. The plum1 module, composed of 58 genes, had a high positive correlation of 0.82 with the MF treatment. Six annotated genes, prophenoloxidase 1, prophenoloxidase 2, prophenoloxidase 3, larval serum protein 1, purine-rich binding protein-alpha, and sodium chloride cotransporter 69 were identified as hub genes (Figure 3D).

### 3.5. Validation of the Transcriptome Results

**Figure 4:**
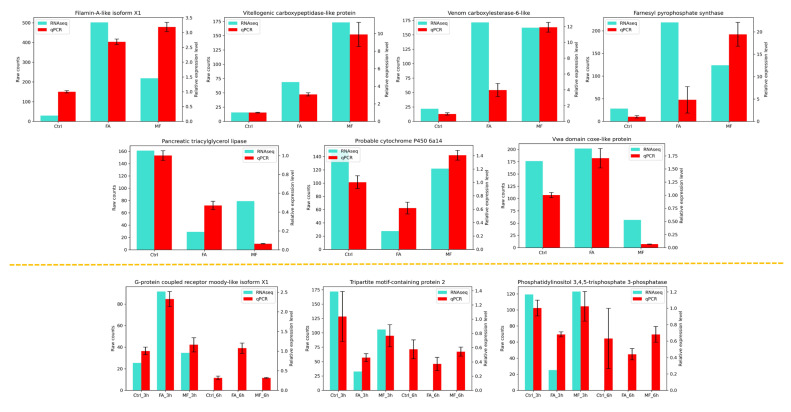
Consistency of RNA-sequencing and qPCR results for 10 randomly chosen DEGs.

14 DEGs identified in the FA or MF were randomly selected for qPCR validation, including Filamin-A-like isoform X1, Vitellogenic carboxypeptidase-like protein, Venom carboxylesterase-6-like, Farnesyl pyrophosphate synthase, Pancreatic triacylglycerol lipase, Probable cytochrome P450 6a14, Vwa domain coxe-like protein, phosphatidylinositol 3,4,5-trisphosphate 3-phosphatase, G-protein coupled receptor moody-like isoform X1, tripartite motif-containing protein 2, cation-independent mannose-6-phosphate receptor, copper-transporting ATPase 1, E3 ubiquitin-protein ligase HUWE1, and polyubiquitin-B. As shown in Figure 4 and Figures S1, 10 out of the 14 qPCR results were consistent with transcriptomic analyses results.

## 4. Discussion

Hepatopancreas, also known as the digestive gland or the midgut gland, is important for immune functions, metabolisms, and vitellogenesis in crustaceans [27,28]. In decapod crustaceans, sesquiterpenoid hormones were suggested to be regulators in hepatopancreas and sex organs [29]. In our sesquiterpenoid hormone FA treatment of the female hepatopancreas, expression of farnesyl pyrophosphate (FPPS) was induced and upregulated from a low expression level (Figure 2C). FPPS takes part in the conversion of farnesyl diphosphate into farnesol, which is a precursor present during the synthesis of FA. This enzyme involved in the biosynthesis of isoprenoids has been found to play an important role in ovarian development [30], and knockdown of FPPS in *N. denticulata* has also been shown to influence the expression of vitellogenin [31]. Our data suggested that FA could potentially be involved in the regulation of vitellogenesis in shrimp *Neocaridina*, which is in agreement with previous studies on other crustaceans [18,19].

Similar but unlike the situations in mammals where the liver and pancreas separately function to produce digestive enzymes, hepatopancreas is a united organ in decapod crustaceans that synthesizes and secretes digestive enzymes for the final digestion of ingested food before their subsequent intake into the body [32,33]. Here, we also showed that FA could cause the upregulation of *carboxypeptidase*, *trypsin*, and *pancreatic lipase* gene expression. Carboxypeptidase is a protease that cleaves the C-terminus of the peptide chain and releases amino acids [34]. Both carboxypeptidase A, which is responsible for the cleavage of aliphatic residues, and carboxypeptidase B, which is responsible for the cleavage of basic amino residues, were both upregulated after FA treatment. Trypsin is another key protease for food digestion in shrimps [35], and pancreatic lipase can also help with digestion. Our study suggested that FA could aid in shrimp growth by promoting the conversion and absorption of food nutrients.

Intriguingly, we have also revealed that the expressions of both hemocyanin-like isoform X1 and hemocyanin A chain-like genes were upregulated in hepatopancreas after the MF treatment, while chymotrypsin and trypsin genes were also upregulated after FA treatment. Hemocyanin is the copper-binding protein and has been demonstrated to be involved in the immune responses in various decapod crustaceans [36]. On the other hand, hemocyanin expression could be activated under the digestion of trypsin or chymotrypsin [37]. Furthermore, two of the mitogen-activated protein kinase (MAPK) pathway genes, *calmodulin* and *P-type Cu+ transporter*, were also found to be downregulated after the FA treatment in the hepatopancreas, where MAPK has been found to be involved in diverse biological processes in arthropods, including cold stress [38], cell proliferation and apoptosis [39], and resistance to pathogens [40].

WGCNA is frequently used to identify the specific effects of treatment on genes [41,42], and it was used to reveal the effects of FA on hepatopancreas here. Among the identified hub genes, prophenoloxidase has been shown to be a crucial element of a crustacean’s innate immune system and to resist pathogens [43], and it could be triggered by the steroid hormone 20-hydroxy-ecdysone (20 E) [43] for a crustacean’s development [44]. Another hub gene larval serum protein 1 served as amino acid storage during metamorphosis [45,46], and it could also be regulated by 20 E [47]. This analysis strongly suggests that FA is involved in the development and immune functions of crustaceans.

Despite that the method by which the two sesquiterpenoid hormones cooperate to function in the regulation of immune responses remains to be elucidated, the bottom line is that MF and FA regulate different sets of genes in decapod crustaceans. It is perhaps a good time to reinvestigate the neglected functional roles of the FA in crustaceans, as well as in other invertebrates [12,48].

## 5. Conclusions

The first investigation of the transcriptomic responses of female shrimp hepatopancreas after treatments of FA and MF has allowed us to reveal the differential gene sets regulated by these two sesquiterpenoid hormones. In the last three decades, MF has been the key candidate to understand how the sesquiterpenoid hormone functions in crustaceans—and it is now time to reinvestigate the functions of FA in crustaceans.

## Figures and Tables

**Table 1:** Statistics of RNA sequencing data.

Sample	Replicates	Clean Reads	Clean Bases	Q30%	Q20%	GC%	Accession Number
CT	CT1	44,726,374	6,708,865,897	94.17	98.04	41.99	SAMN43294670
CT	CT2	35,940,474	5,391,000,460	94.18	98.02	42.36	SAMN43294671
CT	CT3	39,464,592	5,919,608,543	93.97	97.94	42.85	SAMN43294672
FA	FA1	42,974,740	6,446,117,636	94.17	98.03	42.12	SAMN43294673
FA	FA2	40,680,348	6,101,970,714	93.97	97.97	41.71	SAMN43294674
FA	FA3	38,650,158	5,797,452,168	93.58	97.79	42.56	SAMN43294675
MF	MF1	42,207,424	6,331,034,015	94.34	98.11	41.78	SAMN43294676
MF	MF2	37,425,622	5,613,776,825	94.24	98.05	42.41	SAMN43294677
MF	MF3	37,352,120	5,602,746,352	94.14	98.04	41.68	SAMN43294678

## Data Availability

The raw reads generated in this study have been deposited into the NCBI database under the BioProject accession number PRJNA1150739.

## Supplementary Materials

The following supporting information can be downloaded at: https://www.mdpi.com/article/10.3390/biom15060815/s1, Figure S1: Inconsistency of RNA sequencing and qPCR results for 3 randomly chosen genes.; Table S1: qPCR primers used in this study; Table S2: List of DEGs and their raw counts in FA treatment group; Table S3: List of DEGs and their raw counts in MF treatment group; Table S4: GO pathway enrichment analysis of DEGs between control and FA treatment groups; Table S5: GO pathway enrichment analysis of DEGs between control and MF treatment groups.
