# Supplementary figures and images for: Sesquiterpenoid Hormones Farnesoic Acid and Methyl Farnesoate Regulate Different Gene Sets in Shrimp Neocaridina davidi Hepatopancreas
Source: Biomolecules. 2025 Jun 4;15(6):815. doi: 10.3390/biom15060815 (PMC12190968; doi:10.3390/biom15060815)

## Slide 1
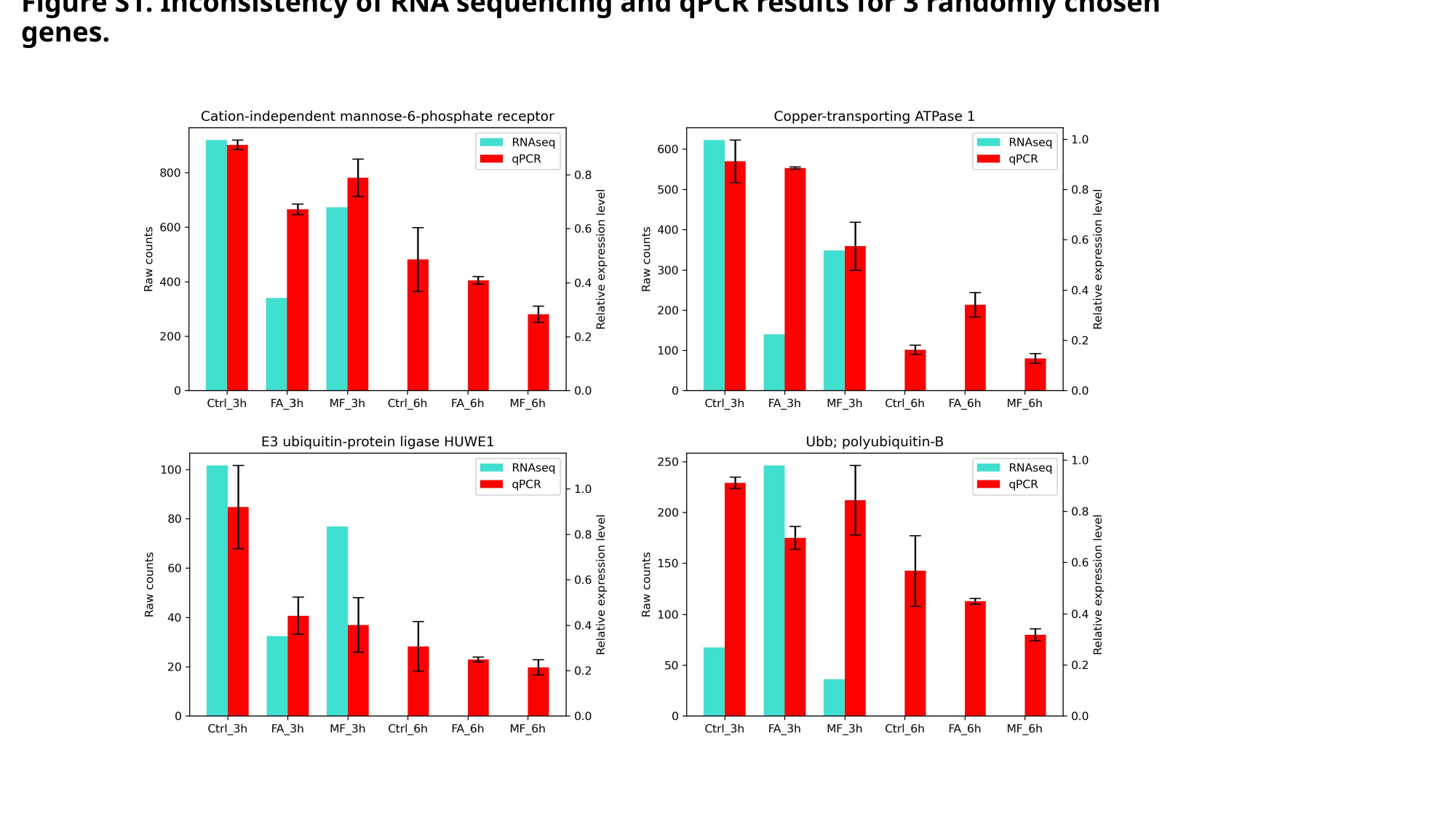

Figure S1. Inconsistency of RNA sequencing and qPCR results for 3 randomly chosen genes.

Supplement: Supplementary file 1 [file biomolecules-15-00815-s001.zip › Supplementary figure.pptx]
